# Comparative Clinical Outcomes of Non-surgical and Surgical Treatment Methods for Lumbar Canal Stenosis: A Systematic Review

**DOI:** 10.7759/cureus.111963

**Published:** 2026-07-02

**Authors:** Siddhi Patrekar, Sandeep Shinde, Aishwarya Shinde, Durga Jagdale, Nidhi Shetty

**Affiliations:** 1 Department of Musculoskeletal Sciences, Krishna College of Physiotherapy, Krishna Vishwa Vidyapeeth (Deemed to be University), Karad, IND; 2 Department of Orthopedic Manual Physiotherapy, Krishna College of Physiotherapy, Krishna Vishwa Vidyapeeth (Deemed to be University), Karad, IND

**Keywords:** conservative treatment, decompression surgery, functional outcomes, lumbar canal stenosis, lumbar spinal stenosis, neurogenic claudication, physiotherapy, systematic review

## Abstract

Lumbar canal stenosis (LCS) is a common degenerative spinal disorder that causes narrowing of the spinal canal, leading to low back pain, radicular symptoms, neurogenic claudication, impaired walking ability, and reduced quality of life. This systematic review aimed to compare the clinical outcomes of non-surgical and surgical treatment methods for patients with LCS.

A comprehensive literature search was conducted across PubMed, Scopus, Web of Science, Cochrane Library, and Google Scholar for studies published between 2005 and 2026. Following PRISMA 2020 guidelines, 22 studies met the inclusion criteria, comprising randomized controlled trials, cohort studies, retrospective studies, and secondary analyses.

Eleven studies evaluated non-surgical interventions, including physiotherapy, exercise therapy, manual therapy, multimodal rehabilitation, and acupuncture, while 11 studies assessed surgical interventions, including decompression surgery, endoscopic decompression, fusion procedures, and interspinous devices. Among the 22 included studies, 19 reported clinical outcomes, of which 16 demonstrated significant improvements in pain, disability, walking capacity, and functional status. Seven non-surgical studies reported reductions in pain and disability with improved walking performance following physiotherapy, exercise, manual therapy, multimodal rehabilitation, or acupuncture. Eight surgical studies demonstrated clinically meaningful improvements in pain relief, functional status, and walking ability, with comparative studies consistently favoring surgical treatment over conservative management for short- and long-term outcomes. Non-surgical interventions were effective in reducing symptoms and enhancing quality of life, particularly in patients with mild-to-moderate disease severity. However, surgical decompression generally produced greater and more durable improvements in pain relief, functional recovery, and walking ability, especially among patients with persistent neurogenic claudication or inadequate response to conservative management. Endoscopic decompression offered additional advantages of reduced blood loss and faster postoperative recovery, whereas fusion surgery showed no significant benefit over decompression alone.

Overall, the evidence suggests that both treatment modalities have important roles in the management of LCS, with treatment selection depending on symptom severity, functional impairment, patient preferences, and clinical presentation. Several studies reported greater and more sustained improvements following surgical intervention; however, substantial heterogeneity among the included studies limits definitive conclusions regarding comparative effectiveness. Conservative management remains an important first-line treatment option, while surgical intervention may be beneficial for appropriately selected patients.

## Introduction and background

Lumbar canal stenosis (LCS) is a prevalent degenerative spinal disorder characterized by narrowing of the spinal canal, resulting in compression of neural and vascular structures within the lumbar spine. It is one of the most common causes of pain, disability, and reduced mobility among older adults. Patients frequently present with low back pain, radicular leg pain, and neurogenic intermittent claudication, which significantly impair walking capacity, physical function, and quality of life [1]. The prevalence of LCS increases with age due to progressive degenerative changes such as intervertebral disc degeneration, facet joint hypertrophy, osteophyte formation, and ligamentum flavum thickening [2].

The management of LCS remains a significant clinical challenge because symptom severity, functional impairment, and radiological findings often vary among patients. Current treatment options can be broadly categorized into non-surgical and surgical approaches. Non-surgical management commonly includes physiotherapy, exercise therapy, patient education, activity modification, pharmacological treatment, and epidural steroid injections. These interventions aim to reduce pain, improve function, and enhance walking tolerance while avoiding the risks associated with surgery. However, the effectiveness of conservative treatment may be limited in patients with severe neurological compromise or persistent neurogenic claudication.

Non-surgical management is generally considered the first-line treatment for patients with mild-to-moderate LCS, particularly in the absence of progressive neurological deficits or severe functional impairment. Patient selection for conservative treatment should be individualized and based on symptom severity, degree of disability, imaging findings, response to previous treatment, comorbidities, and patient preferences.

Surgical treatment, primarily involving decompressive procedures with or without spinal fusion, is generally recommended for patients who fail to respond adequately to conservative management or who experience progressive neurological deficits. Evidence from the Spine Patient Outcomes Research Trial (SPORT) demonstrated that surgical intervention resulted in greater improvements in pain relief, physical function, and disability scores compared with non-operative treatment in patients with symptomatic lumbar spinal stenosis [3]. Furthermore, long-term follow-up data indicated that many of these benefits were maintained for up to eight years following surgery [4].

Advances in surgical techniques have focused on minimizing tissue damage while achieving adequate neural decompression. Microsurgical bilateral decompression through a unilateral approach has been associated with favorable clinical outcomes and improved postoperative function in patients with LCS [5]. Nevertheless, variations in surgical practice patterns across different healthcare systems have been reported, suggesting that treatment decisions may be influenced by factors beyond clinical presentation alone [6].

Several prognostic factors may affect treatment outcomes. Preoperative MRI characteristics have been shown to predict postoperative clinical improvement, highlighting the importance of radiological assessment in patient selection and treatment planning [7]. Additionally, spinal alignment and sagittal balance have emerged as important considerations in determining the suitability and success of surgical interventions [8].

Previous systematic reviews and major clinical trials have evaluated the effectiveness of specific surgical or non-surgical interventions for LCS. However, many reviews focused on individual treatment modalities, specific patient populations, or limited outcome measures, and several were conducted before the publication of recent studies evaluating contemporary surgical techniques, rehabilitation strategies, and minimally invasive interventions. Furthermore, direct comparison of surgical and non-surgical treatment outcomes across multiple clinical domains remains limited. Therefore, this systematic review aims to provide an updated and comprehensive synthesis of the current evidence by evaluating and comparing the clinical outcomes of both surgical and non-surgical management approaches for LCS.

Although numerous studies have evaluated the effectiveness of both non-surgical and surgical treatments for LCS, the relative benefits and limitations of these approaches remain a subject of ongoing debate. Differences in study design, patient populations, outcome measures, and follow-up durations have contributed to inconsistencies in the literature. Although previous systematic reviews have evaluated either surgical or conservative interventions for lumbar spinal stenosis, many focused on specific treatment modalities, such as decompression surgery, fusion procedures, exercise therapy, or physiotherapy, rather than providing a comprehensive comparison of both treatment approaches within a single review. Furthermore, recent evidence from randomized controlled trials and observational studies evaluating minimally invasive surgical techniques, multimodal rehabilitation programs, and acupuncture has not been synthesized alongside traditional treatment strategies. Therefore, there remains a need for an updated systematic review that integrates contemporary evidence and directly compares the clinical outcomes of both non-surgical and surgical management. This review addresses this gap by synthesizing evidence from studies published between 2005 and 2026 and evaluating pain, disability, walking capacity, neurogenic claudication symptoms, and functional outcomes across both treatment approaches to support evidence-based clinical decision-making. It aims to systematically evaluate and compare the clinical outcomes of non-surgical and surgical treatment methods in patients with LCS, focusing on pain, functional disability, walking capacity, and neurogenic claudication. The objective of this systematic review was to evaluate and compare the clinical outcomes of surgical and non-surgical treatment approaches for LCS and to synthesize current evidence to support evidence-based clinical decision-making.

## Review

Methedology 

Study Design

This systematic review was conducted to compare the clinical outcomes of surgical and non-surgical treatment methods for LCS. The review followed the Preferred Reporting Items for Systematic Reviews and Meta-Analyses (PRISMA 2020) guidelines to ensure a transparent and structured approach to study identification, selection, data extraction, and synthesis. Randomized controlled trials, randomized clinical trials, cohort studies, retrospective studies, and secondary analyses evaluating treatment outcomes in patients with LCS were considered for inclusion.

Search Strategy

A comprehensive literature search was conducted in electronic databases including PubMed, Scopus, Web of Science, Cochrane Library, and Google Scholar. The search was performed for studies published from January 2005 to June 2026. The literature search was conducted between May and June 2026, and the final search was completed on 15 June 2026. Studies published from January 2005 through June 2026 were considered for inclusion. The evidence cut-off date for study inclusion was 15 June 2026; therefore, only articles available in the searched databases up to this date were eligible for review. Medical Subject Headings (MeSH) terms and keywords were combined using Boolean operators. The search terms included: 'Lumbar canal stenosis', 'Lumbar spinal stenosis', 'Neurogenic claudication', 'Surgical treatment', 'Decompression surgery', 'Fusion surgery', 'Endoscopic decompression', 'Physical therapy', 'Physiotherapy', 'Exercise therapy', 'Conservative treatment', 'Non-surgical management', and 'Clinical outcomes'. The search strategy was adapted for each database, and the complete database-specific search strings are provided in Appendix 1 in accordance with PRISMA 2020 recommendations. Additionally, reference lists of eligible studies were manually screened to identify relevant articles that may have been missed during the electronic search.

Inclusion Criteria

Studies were included in this systematic review if they involved adult patients (≥18 years) diagnosed with LCS and evaluated either surgical or non-surgical treatment interventions. Eligible study designs included randomized controlled trials, randomized clinical trials, cohort studies, retrospective studies, and secondary analyses of randomized trials. Studies were required to report at least one relevant clinical outcome, including pain intensity, functional disability, walking performance, neurogenic claudication symptoms, quality of life, or patient satisfaction. Only full-text articles published in peer-reviewed journals and written in English between 2005 and 2026 were considered for inclusion.

Exclusion Criteria

Studies were excluded if they focused on cervical or thoracic spinal stenosis, involved animal or cadaveric models, or primarily investigated diagnostic methods, imaging findings, or surgical techniques without reporting clinical outcomes. Case reports, case series, narrative reviews, systematic reviews, meta-analyses, editorials, conference abstracts, letters to the editor, and expert opinion articles were also excluded. Additionally, studies with insufficient outcome data, duplicate publications, or those not available in English were not considered for inclusion in the review.

Study Selection

Titles and abstracts were screened against the predefined eligibility criteria, followed by full-text assessment of potentially relevant articles. Any uncertainties regarding study eligibility were resolved through discussion among the reviewers.

Data Extraction

Data extracted from eligible studies included author details, publication year, study design, sample size, interventions, outcome measures, and key findings. Any discrepancies in data extraction were resolved through discussion among the reviewers.

Quality Assessment

The methodological quality and risk of bias of the included studies were assessed according to the study design. Randomized controlled trials and randomized clinical trials were evaluated using the Revised Cochrane Risk of Bias Tool (RoB 2), which assesses bias arising from the randomization process, deviations from intended interventions, missing outcome data, outcome measurement, and selective reporting [9]. Non-randomized intervention studies were assessed using the Risk of Bias in Non-randomized Studies of Interventions (ROBINS-I) tool, which evaluates potential bias related to confounding, participant selection, intervention classification, deviations from intended interventions, missing data, outcome measurement, and reporting bias [10]. Observational and cohort studies were appraised using the Newcastle-Ottawa Scale (NOS), which assesses study quality based on participant selection, comparability of study groups, and outcome assessment. The results of these assessments were considered during data synthesis and interpretation of the review findings [11].

Data Synthesis

Due to substantial heterogeneity in study designs, interventions, outcome measures, follow-up durations, and reporting methods, a narrative synthesis was performed. Quantitative meta-analysis was not considered appropriate because pooling of data could have produced misleading estimates of treatment effects.

**Figure 1:**
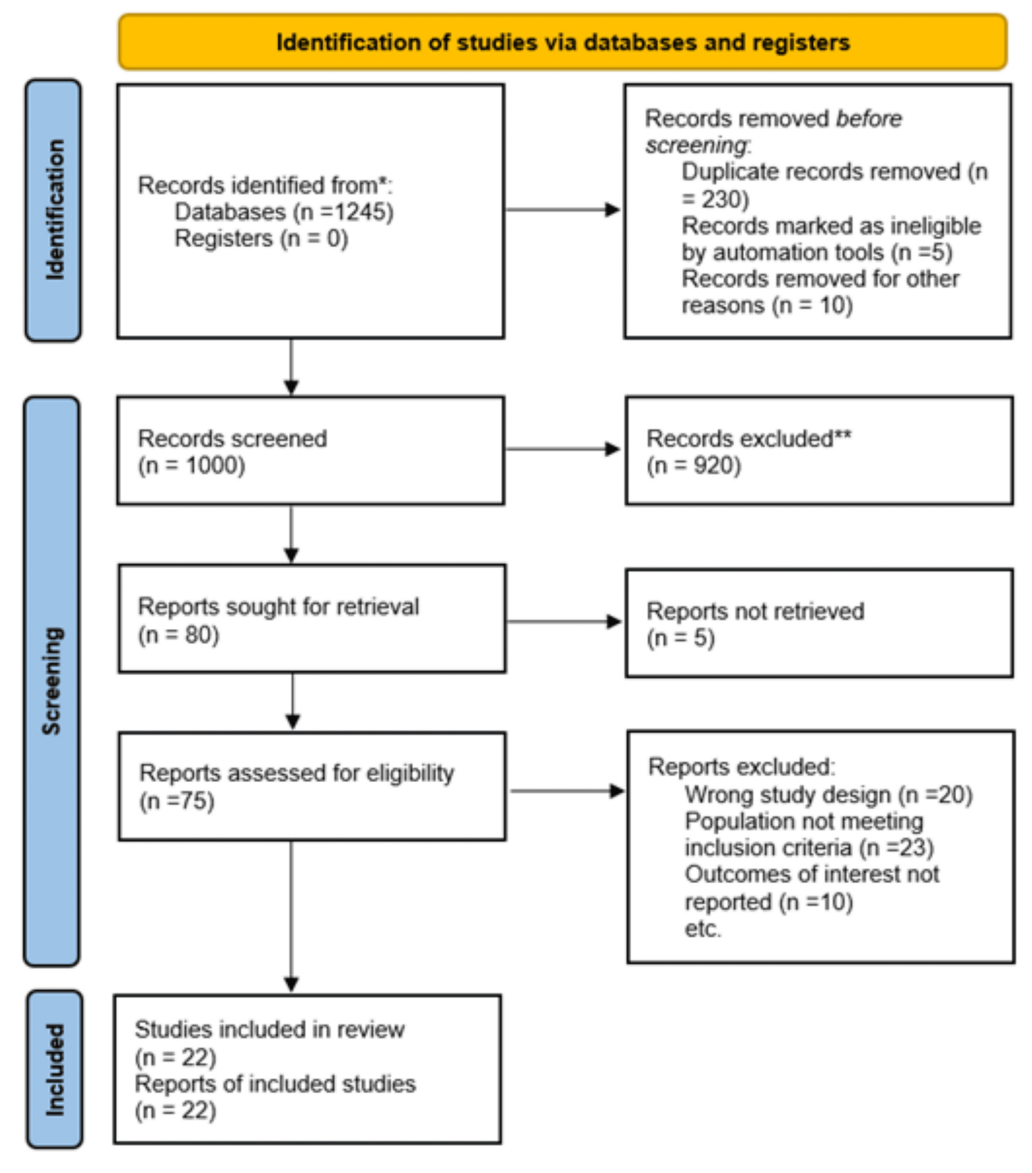
Preferred Reporting Items for Systematic Reviews and Meta-Analyses (PRISMA) Flowchart

A total of 1,245 records were identified through database searching. After removing duplicates and other ineligible records, 1,000 studies were screened. Following title and abstract screening, 75 full-text articles were assessed for eligibility. Fifty-three studies were excluded because of inappropriate study design, ineligible population, or failure to report relevant outcomes. Ultimately, 22 studies were included in the systematic review, as given in Figure 1.

Seven studies evaluating non-surgical interventions for lumbar spinal stenosis were included in the evidence synthesis. The interventions comprised manual therapy, exercise therapy, physiotherapy, multimodal rehabilitation, acupuncture, and minimally invasive lumbar decompression procedures. Overall, these studies demonstrated improvements in pain, disability, walking capacity, neurogenic claudication symptoms, and functional outcomes, supporting the role of conservative management as an effective treatment option for selected patients with lumbar spinal stenosis, as given in Table 1.

**Table 1:** Non-surgical Studies Included in Evidence Synthesis RCT: Randomized Controlled Trial; ODI: Oswestry Disability Index; MSSS: Modified Swiss Spinal Stenosis Questionnaire; NRS: Numerical Rating Scale; SF-36: Short Form-36 Health Survey; JOA: Japanese Orthopaedic Association Score; VAS: Visual Analog Scale

Author (Year)	Study Design	Intervention	Sample Size	Outcomes Assessed	Main Findings
Shah et al., 2025 [12]	Clinical Trial	Physiotherapy program	60	Claudication symptoms, function	Improved functional outcomes
Roseen et al., 2025 [13]	Secondary RCT Analysis	Non-surgical treatment	259	Pain, disability	Improved patient-reported outcomes
Zhu et al., 2024 [14]	RCT	Acupuncture	196	MSSS, NRS, ODI	Significant reduction in pain and disability
Zhong et al., 2023 [15]	Randomized Clinical Trial	Gait-analysis-based postoperative exercise vs conventional exercise	60	VAS, ODI, JOA, spatiotemporal gait parameters, walking performance, and functional mobility measures	Gait-based exercise demonstrated greater improvement in walking performance and functional mobility compared with conventional postoperative exercise
Deer et al., 2021 [16]	RCT	mild® procedure	131	Pain, walking tolerance	Improved mobility and symptoms
Ammendolia & Chow, 2015 [17]	Retrospective Study	Multimodal rehabilitation	49	Walking capacity, function	Improved neurogenic claudication symptoms
Whitman et al., 2006 [18]	RCT	Manual therapy + exercise + treadmill walking	58	ODI, walking capacity	Improved disability and walking function
Delitto et al., 2015 [19]	RCT	Physical therapy	169	SF-36, ODI	Significant improvement with conservative treatment

Five studies identified during the literature search were protocol publications that did not report clinical outcome data at the time of review. Although these studies met the eligibility criteria for inclusion, they were excluded from the evidence synthesis evaluating treatment effectiveness because outcome results were unavailable. These studies were retained for descriptive reporting to acknowledge ongoing and planned research on surgical and non-surgical interventions for lumbar spinal stenosis, as given in Table 2.

**Table 2:** Protocol Studies Excluded From Evidence Synthesis RCT: Randomized Controlled Trial

Author (Year)	Study Design	Planned Sample Size	Intervention	Reason for Exclusion
Zhou et al., 2020 [20]	RCT Protocol	80	Acupuncture versus sham acupuncture	No clinical outcome data available
Hermansen et al., 2017 [21]	RCT Protocol	437	Three decompression techniques	No clinical outcome data available
Schneider et al., 2014 [22]	RCT Protocol	180	Non-surgical treatment approaches	No clinical outcome data available
Marchand et al., 2015 [23]	RCT Protocol	130	Prehabilitation before surgery	No clinical outcome data available
Moojen et al., 2010 [24]	RCT Protocol	200	Interspinous implant vs decompression	No clinical outcome data available

Included studies of surgical interventions for lumbar spinal stenosis with neurogenic claudication. The included studies assessed decompression surgery, endoscopic and microscopic decompression techniques, decompression with or without fusion, interspinous devices, and surgery compared with conservative treatment. Most studies reported significant improvements in pain, disability, walking capacity, and functional outcomes following surgery. Decompression surgery consistently demonstrated favorable short- and long-term results, while endoscopic techniques provided additional minimally invasive benefits such as reduced blood loss and faster recovery. Studies comparing fusion with decompression alone found no added benefit of fusion, and conventional decompression generally outperformed interspinous devices. Overall, the evidence suggests that decompressive surgery is an effective treatment for symptomatic lumbar spinal stenosis, particularly in patients with persistent neurogenic claudication and functional limitations, as given in Table 3.

**Table 3:** Summary of Included Studies Assessing Surgical Treatment Outcomes in Lumbar Spinal Stenosis. VAS: Visual Analog Scale; ODI: Oswestry Disability Index; EQ-5D: EuroQol Five Dimension Questionnaire; 6MWD: 6 Minute Walk Distance; 6MWT: 6 Minute Walk Test; JOA: Japanese Orthopaedic Association Score; PGIC: Patient Global Impression of Change; SF-36: Short Form 36 Health Survey; RMDQ: Roland-Morris Disability Questionnaire.

Author & Year	Study Design	Sample Size	Pain Type (Localized / Radicular)	Neurogenic Claudication	Outcome Measures	Surgery	Intervention	Results	Conclusion
Minetama et al., 2026 [25]	Secondary Analysis of a Randomized Controlled Trial	86	Localized and radicular symptoms	Present	Physical function measures, patient-reported outcomes	Lumbar decompression surgery	Postoperative rehabilitation	Improvements in physical function were associated with better patient-reported outcomes following surgery	Postoperative rehabilitation and recovery of physical function positively influence patient-reported outcomes after lumbar stenosis surgery
Kotheeranurak et al., 2023 [26]	Randomized Controlled Trial	60	Radicular leg pain with/without low back pain	Present	VAS, ODI, EQ-5D, Modified MacNab criteria	Endoscopic decompression vs microscopic decompression	Full-endoscopic decompression vs tubular microscopic decompression	Both groups improved significantly in pain, ODI, and walking ability; endoscopic group had less blood loss and faster recovery	Both techniques are effective; endoscopic surgery offers minimally invasive advantages
Takenaka et al., 2022 [27]	Prospective Observational Study	84	Localized and radicular symptoms	Present	ODI, VAS, 6MWD, JOA, PGIC	Lumbar decompression surgery	Assessment of 6-minute walk distance responsiveness after surgery	Significant postoperative improvement in walking distance and functional status	6MWD is a valid measure of recovery after lumbar stenosis surgery
Rodrigues & Natour, 2021 [28]	Single-blinded Randomized Controlled Trial	50	Radicular pain predominant	Present	ODI, VAS, SF-36, Walking capacity assessment	Lumbar decompression surgery	Surgery plus rehabilitation vs rehabilitation alone	Surgical group showed greater improvements in pain, disability, and walking capacity	Surgery provides superior outcomes in appropriately selected patients
Försth et al., 2016 [29]	Randomized Controlled Trial	247	Low back pain and radicular symptoms	Present	ODI, VAS, EQ-5D, 6MWT	Decompression alone or decompression with fusion	Fusion surgery vs decompression alone	No significant differences in disability, pain, or walking outcomes between groups	Fusion did not provide additional benefit over decompression alone
Slätis et al., 2011 [30]	Long-term Randomized Controlled Trial Follow-up	94	Radicular leg pain with low back pain	Present	VAS, ODI, Walking capacity, Patient-reported functional status	Decompressive surgery	Surgical vs conservative treatment	Surgical benefits persisted over long-term follow-up with improved pain and function	Surgery provides durable long-term symptom relief
Malmivaara et al., 2007 [31]	Randomized Controlled Trial	94	Radicular leg pain and low back pain	Present	ODI, VAS, Walking ability status	Decompressive surgery	Surgery vs nonoperative management	Surgery produced greater reductions in pain and disability and improved walking capacity	Surgery offers superior short-term outcomes compared with conservative care
Atlas et al., 2005 [32]	Prospective Cohort Study (Maine Lumbar Spine Study)	148 surgical, 67 nonsurgical	Radicular pain and low back pain	Present	Pain severity scale, RMDQ, Symptom improvement scale, Functional status measures	Lumbar decompression surgery	Surgical vs nonsurgical management	Surgical patients maintained greater improvement in pain, function, and satisfaction at 8–10 years	Long-term outcomes generally favor surgical management for symptomatic lumbar stenosis

Assessment of Risk of Bias

To ensure the credibility of the synthesized data, a risk-of-bias evaluation was performed for each selected study. The reliability of the reported outcomes was confirmed by rigorously evaluating all selected literature using validated research tools.

The RoB 2 assessment showed that most included studies had an overall rating of some concerns, mainly due to limitations in the randomization process and selective reporting of results. The domains of missing outcome data and outcome measurement were generally assessed as low risk of bias. Zhu et al. (2024) was the only study rated as having an overall low risk of bias across all domains. Protocol studies were rated as having some concerns because complete outcome data were not available [15]. Overall, the included studies demonstrated moderate to good methodological quality, as given in Table 4.

**Table 4:** Risk of Bias Assessment of Non-surgical Studies Using the RoB 2 tool Some concerns means that there is insufficient information or a potential risk of bias in one or more domains, but the issues are not serious enough to classify the study as having a high risk of bias. [9]

Study	Randomization Process	Deviations from Intended Interventions	Missing Outcome Data	Measurement of Outcome	Selection of Reported Result	Overall Risk of Bias
Roseen et al., 2025 [13]	Some Concerns	Low Risk	Low Risk	Low Risk	Some Concerns	Some Concerns
Zhu et al., 2024 [14]	Low Risk	Low Risk	Low Risk	Low Risk	Low Risk	Low Risk
Deer et al., 2021 [16]	Low Risk	Some Concerns	Low Risk	Low Risk	Some Concerns	Some Concerns
Zhou et al., 2020 [20]	Some Concerns	Some Concerns	Some Concerns	Some Concerns	Some Concerns	Some Concerns
Hermansen et al., 2017 [21]	Some Concerns	Some Concerns	Some Concerns	Some Concerns	Some Concerns	Some Concerns
Delitto et al., 2015 [19]	Some Concerns	Some Concerns	Low Risk	Low Risk	Some Concerns	Some Concerns
Schneider et al., 2014 [22]	Some Concerns	Some Concerns	Some Concerns	Some Concerns	Some Concerns	Some Concerns
Whitman et al., 2006 [18]	Some Concerns	Some Concerns	Low Risk	Low Risk	Some Concerns	Some Concerns
Moojen et al., 2013 [24]	Low Risk	Some Concerns	Low Risk	Low Risk	Low Risk	Some Concerns

NOS was used for assessment of the non-randomized study by Shah et al. (2025) [13]. The study received a total score of 6 out of 9, with good ratings in the selection and outcome domains but no points for comparability. Based on the NOS criteria, the study was classified as having fair methodological quality, indicating a moderate risk of bias and acceptable overall reliability of the findings, as given in Table 5.

**Table 5:** Newcastle-Ottawa Scale Quality Assessment of Non-surgical Studies [11]

Author	Selection (max 4)	Comparability (max 2)	Outcome (max 3)	Total Score (0–9)	Overall Rating
Shah et al. (2025) [12]	3	0	3	6	Fair

ROBINS-I assessment for the non-randomized study by Ammendolia and Chow (2015) [20]. The study demonstrated a serious risk of bias overall, primarily due to concerns related to confounding, which may have influenced the observed treatment effects. Moderate risk was identified in participant selection, deviations from intended interventions, missing data, outcome measurement, and selective reporting, while intervention classification was rated as low risk. Overall, the findings should be interpreted with caution due to the potential influence of bias, as given in Table 6.

**Table 6:** Risk of Bias Assessment of Non-surgical Studies Using the Risk of Bias in Non-randomized Studies of Interventions (ROBINS-I) Tool [10]

Author	Confounding	Selection of Participants	Classification of Intervention	Deviations from Intended Intervention	Missing Data	Measurement of Outcomes	Selection of Reported Results	Overall ROBINS-I Judgment
Ammendolia & Chow (2015) [17]	Serious	Moderate	Low	Moderate	Moderate	Moderate	Moderate	Serious Risk

RoB 2 assessment of randomized studies evaluating surgical interventions for lumbar spinal stenosis with neurogenic claudication. Most studies were judged as having some concerns overall, mainly due to limitations in the randomization process, deviations from intended interventions, and selective reporting of results. The domains of missing outcome data and measurement of outcomes were generally rated as low risk, indicating adequate follow-up and reliable outcome assessment. Protocol studies were rated as having some concerns across all domains because complete outcome data were unavailable. Overall, the included studies demonstrated acceptable methodological quality, although moderate concerns regarding bias remain in several trials, as given in Table 7.

**Table 7:** Risk of Bias Assessment of Included Surgical Studies Using the RoB 2 Tool Some concerns means that there is insufficient information or a potential risk of bias in one or more domains, but the issues are not serious enough to classify the study as having a high risk of bias. [9]

Study	Randomization Process	Deviations from Intended Interventions	Missing Outcome Data	Measurement of Outcome	Selection of Reported Result	Overall Risk of Bias
Minetama et al., 2026 [25]	Some Concerns	Low Risk	Low Risk	Low Risk	Some Concerns	Some Concerns
Kotheeranurak et al., 2023 [26]	Low Risk	Some Concerns	Low Risk	Low Risk	Some Concerns	Some Concerns
Rodrigues & Natour, 2021 [28]	Some Concerns	Some Concerns	Low Risk	Low Risk	Some Concerns	Some Concerns
Försth et al., 2016 [29]	Low Risk	Some Concerns	Low Risk	Low Risk	Low Risk	Some Concerns
Slätis et al., 2011 [30]	Some Concerns	Some Concerns	Low Risk	Low Risk	Some Concerns	Some Concerns
Malmivaara et al., 2007 [31]	Some Concerns	Some Concerns	Low Risk	Low Risk	Some Concerns	Some Concerns

NOS assessment for two observational studies evaluating surgical outcomes in lumbar spinal stenosis. Takenaka et al. (2022) achieved a score of 6/9 [24], indicating fair methodological quality, while Atlas et al. (2005) received the maximum score of 9/9 [32], reflecting good methodological quality with a low risk of bias. Overall, the observational evidence ranged from fair to high quality, supporting the reliability of the reported findings, as given in Table 8.

**Table 8:** Newcastle-Ottawa Scale Quality Assessment of Surgical Studies [11]

Author	Selection (max 4)	Comparability (max 2)	Outcome (max 3)	Total Score (0–9)	Overall Rating
Takenaka et al. (2022) [27]	3	0	3	6	Fair
Atlas et al. (2005) [32]	4	2	3	9	Good

Discussion

Lumbar spinal stenosis is a common degenerative spinal condition that causes low back pain, radicular symptoms, and neurogenic claudication, leading to significant functional limitations and reduced quality of life. This systematic review compared the clinical outcomes of non-surgical and surgical treatment approaches and found that both strategies can provide meaningful improvements in pain, disability, walking capacity, and overall function. However, the magnitude and durability of benefits varied according to patient characteristics and treatment modality.

Among non-surgical interventions, physiotherapy, exercise therapy, manual therapy, multimodal rehabilitation, and acupuncture demonstrated positive effects on pain reduction and functional recovery. Shah et al. (2025) reported significant improvements in claudication symptoms and functional outcomes following a structured physiotherapy program [13], while Whitman et al. (2006) found that manual therapy combined with exercise and treadmill walking was more effective than exercise alone [22]. Similarly, Zhu et al. (2024) demonstrated that acupuncture significantly improved walking capacity, pain, and disability scores in patients with neurogenic claudication [15]. These findings suggest that conservative treatment remains an effective option, particularly for patients with mild-to-moderate symptoms or those who are not suitable surgical candidates.

The review also highlights the importance of individualized treatment planning. Roseen et al. (2025) showed that specific baseline patient characteristics were associated with greater improvement following nonsurgical management, indicating that patient selection plays a critical role in treatment success [14]. Likewise, multimodal rehabilitation programs demonstrated favorable outcomes in improving walking distance, pain, and disability, emphasizing the value of combining multiple therapeutic approaches to address the complex clinical presentation of lumbar spinal stenosis.

Surgical interventions consistently produced substantial improvements in pain relief, functional status, and walking ability. Randomized trials by Malmivaara et al. (2007), Slätis et al. (2011), and Atlas et al. (2005) reported superior outcomes for surgical treatment compared with conservative management, with benefits persisting over long-term follow-up in many patients. These findings are generally consistent with results from the SPORT trial and other comparative studies, which reported greater improvements in pain and disability following surgical intervention among patients with symptomatic lumbar spinal stenosis. However, the certainty of the evidence should be interpreted cautiously because the present review did not perform a formal Grading of Recommendations Assessment, Development and Evaluation (GRADE) assessment, and the included studies demonstrated heterogeneity in study design, interventions, outcome measures, and follow-up duration. Therefore, while the available evidence suggests potential benefits of surgical intervention in appropriately selected patients, the overall strength of evidence remains variable [30,32].

Advances in minimally invasive surgical techniques have further improved postoperative outcomes. Kotheeranurak et al. (2023) reported comparable clinical improvements between endoscopic and microscopic decompression, with the endoscopic approach offering reduced blood loss and faster recovery. However, not all surgical innovations demonstrated superior outcomes [23]. Försth et al. (2016) found no significant advantage of decompression with fusion compared to decompression alone [26], while Moojen et al. (2013) reported that conventional decompression achieved better outcomes and lower reoperation rates than interspinous process devices [31]. These findings suggest that standard decompression remains the benchmark surgical treatment for many patients with lumbar spinal stenosis.

**Figure 2:**
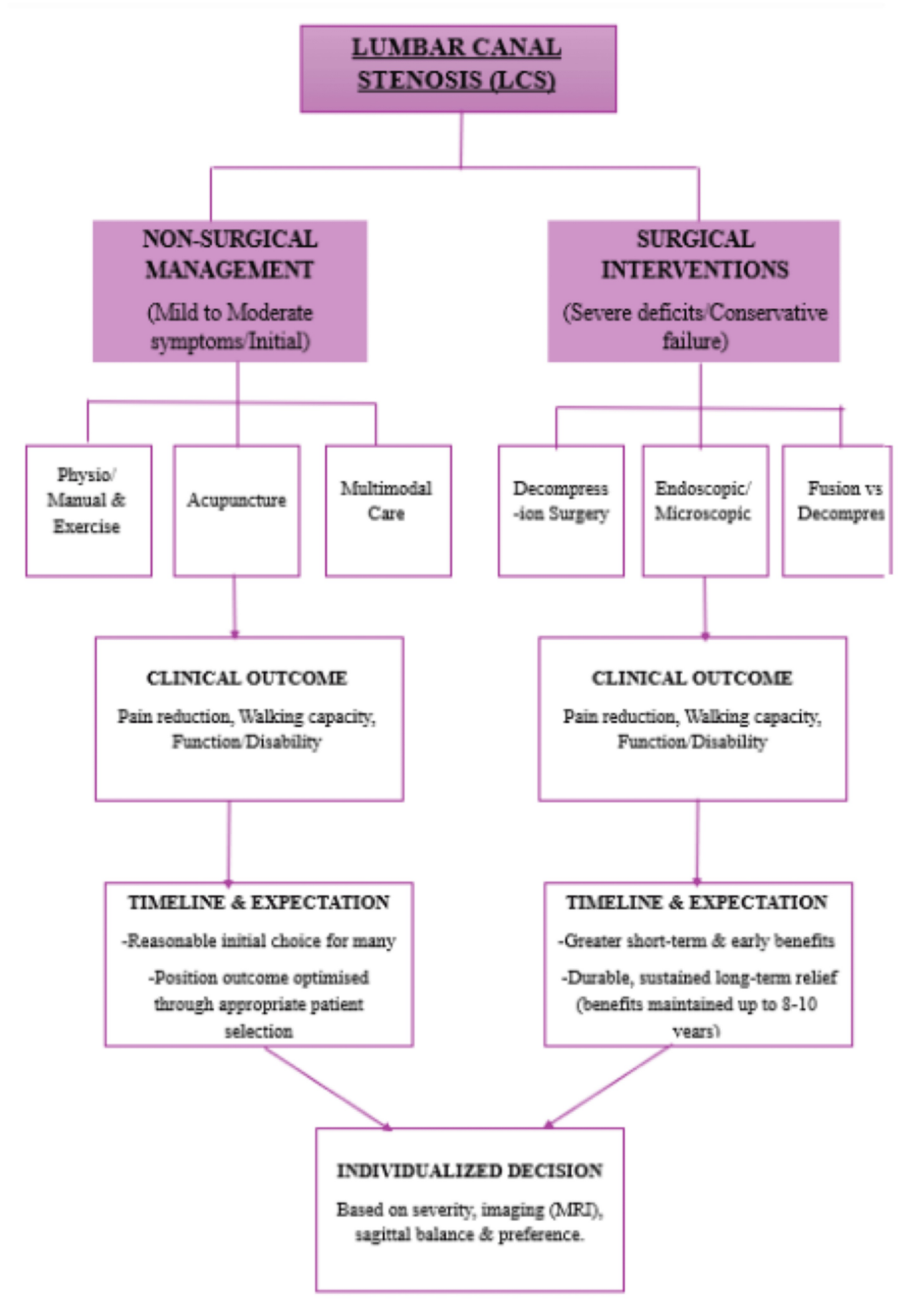
Comparative Overview of Non-surgical and Surgical Treatment Approaches for Lumbar Canal Stenosis This figure was created by the authors using Microsoft Word (Redmond, WA, USA) and is an original illustration.

The treatment pathway identified in this review further emphasizes the complementary roles of non-surgical and surgical management in lumbar spinal stenosis. As illustrated in Figure 2, non-surgical management, including physiotherapy, manual therapy, exercise therapy, acupuncture, and multimodal rehabilitation, is generally considered the initial treatment approach for patients with mild-to-moderate symptoms. These interventions were consistently associated with reductions in pain, improvements in walking capacity, and enhanced functional status. In contrast, surgical interventions are typically reserved for patients with severe neurological deficits, persistent neurogenic claudication, or inadequate response to conservative care. Decompression surgery, endoscopic or microscopic decompression techniques, and fusion procedures demonstrated substantial improvements in pain relief, walking ability, and disability outcomes. The evidence further suggests that surgical treatment may provide greater short-term benefits and more durable long-term symptom relief, with several studies reporting sustained improvements for up to eight to 10 years. Nevertheless, treatment selection should not be based solely on symptom severity but should incorporate clinical presentation, imaging findings, functional impairment, sagittal balance, and patient preferences. Therefore, an individualized decision-making approach remains essential for optimizing outcomes in patients with lumbar spinal stenosis, as given in Figure 2.

The quality assessment demonstrated that most randomized studies had an overall judgment of “some concerns,” mainly related to randomization procedures and selective reporting, while missing outcome data and outcome measurement were generally considered low risk. Zhu et al. (2024) was the only study assessed as having an overall low risk of bias [15]. Observational studies ranged from fair to good quality, whereas the retrospective study by Ammendolia and Chow (2015) was judged to have a serious risk of bias due to confounding [20]. Therefore, although the overall evidence supports the effectiveness of both surgical and non-surgical interventions, the findings should be interpreted considering the methodological limitations of several included studies.

Overall, the findings of this review suggest that non-surgical treatments can effectively improve symptoms and function in many patients with lumbar spinal stenosis, particularly when individualized and delivered through multimodal programs. However, surgical decompression generally provides greater and more sustained improvements in pain, disability, and walking capacity, especially in patients with persistent neurogenic claudication or inadequate response to conservative management. Consequently, treatment decisions should be individualized based on symptom severity, functional impairment, patient preferences, and clinical presentation.

Strengths

This systematic review provides a comprehensive evaluation of both surgical and non-surgical treatment approaches for LCS, offering a broad perspective on current management strategies. The inclusion of randomized controlled trials, observational studies, and prospective studies strengthened the evidence base and enabled a detailed comparison of treatment outcomes. Multiple clinically relevant outcomes, including pain relief, disability, walking capacity, neurogenic claudication symptoms, and quality of life, were assessed, providing a holistic understanding of treatment effectiveness. Furthermore, the methodological quality of the included studies was evaluated using established assessment tools such as RoB 2, ROBINS-I, and the Newcastle-Ottawa Scale, enhancing the credibility and reliability of the review findings. The inclusion of both recent and long-term follow-up studies also enabled an assessment of the sustainability of treatment benefits over time.

Limitations

Despite its strengths, this review has several limitations. Considerable heterogeneity was observed among the included studies in terms of study design, intervention protocols, outcome measures, and duration of follow-up, which limited direct comparison of findings. Several included studies were protocol publications without reported clinical outcomes, reducing the amount of available evidence for certain interventions. Additionally, many randomized studies were judged to have “some concerns” regarding risk of bias, particularly in the domains of randomization and selective reporting. The presence of observational studies, including one study with a serious risk of bias due to confounding, may have influenced the overall strength of the evidence. Variability in patient characteristics and disease severity across studies may further limit the generalizability of the findings. Finally, the absence of a quantitative meta-analysis prevented the calculation of pooled treatment effects and limited the ability to determine the magnitude of benefit associated with specific interventions.

Future Scope

Future research should focus on large multicenter randomized controlled trials with standardized outcome measures and long-term follow-up. Although several studies reported comparable outcomes, substantial heterogeneity in study designs, interventions, outcome measures, and follow-up durations prevented quantitative data pooling in the present review. Therefore, future systematic reviews incorporating meta-analysis and network meta-analysis may provide more precise estimates of the comparative effectiveness of treatment modalities for lumbar canal stenosis.

## Conclusions

Both surgical and non-surgical interventions were associated with improvements in pain, disability, walking capacity, and functional outcomes in patients with LCS. Several studies reported greater and more sustained improvements following surgical decompression, particularly among patients with persistent neurogenic claudication or inadequate response to conservative treatment. However, considerable heterogeneity among the included studies and the absence of pooled quantitative analysis limit definitive conclusions regarding comparative effectiveness. Therefore, treatment decisions should be individualized based on symptom severity, functional limitations, imaging findings, and patient preferences. Further high-quality studies are needed to strengthen the evidence base and guide optimal treatment selection.

## Appendices

Detailed search strategy

PubMed

("lumbar spinal stenosis" OR "lumbar canal stenosis") AND ("neurogenic claudication") AND ("decompression surgery" OR fusion OR physiotherapy OR exercise therapy OR conservative treatment)

Scopus

TITLE-ABS-KEY (("lumbar spinal stenosis" OR "lumbar canal stenosis") AND ("neurogenic claudication") AND ("surgical treatment" OR "physical therapy"))

Web of Science

TS=(("lumbar spinal stenosis" OR "lumbar canal stenosis") AND ("neurogenic claudication") AND ("surgical treatment" OR physiotherapy))

Cochrane Library

("lumbar spinal stenosis" OR "lumbar canal stenosis") AND ("neurogenic claudication") AND ("decompression surgery" OR physiotherapy)

Google Scholar

("lumbar spinal stenosis" OR "lumbar canal stenosis") AND ("surgical treatment" OR physiotherapy OR exercise therapy)

Additional Search Methods

Manual screening of reference lists of included studies.

Removal of duplicate records before screening.

Studies published between January 2005 and June 2026.
